# Next-Generation Sequencing in Lung Cancer Patients: A Comparative Approach in NSCLC and SCLC Mutational Landscapes

**DOI:** 10.3390/jpm12030453

**Published:** 2022-03-13

**Authors:** Cecilia Pop-Bica, Cristina Alexandra Ciocan, Cornelia Braicu, Antonia Haranguș, Marioara Simon, Andreea Nutu, Laura Ancuta Pop, Ondrej Slaby, Atanas G. Atanasov, Radu Pirlog, Nadim Al Hajjar, Ioana Berindan-Neagoe

**Affiliations:** 1 Research Center for Functional Genomics, Biomedicine and Translational Medicine, Iuliu Hatieganu University of Medicine and Pharmacy, 400337 Cluj-Napoca, Romania; cecilia.bica8@gmail.com (C.P.-B.); crisciocan@gmail.com (C.A.C.); braicucornelia@yahoo.com (C.B.); antonia.harangus@yahoo.com (A.H.); andreeanutu.an@gmail.com (A.N.); laura.ancuta.pop@gmail.com (L.A.P.); pirlog.radu@umfcluj.ro (R.P.); 2 Leon Daniello Pulmonology Hospital, 400332 Cluj-Napoca, Romania; simonmariaro@gmail.com; 3 Centre for Molecular Medicine, Central European Institute of Technology, Masaryk University, 62500 Brno, Czech Republic; on.slaby@gmail.com; 4 Department of Comprehensive Cancer Care, Masaryk Memorial Cancer Institute, Faculty of Medicine, Masaryk University, 62500 Brno, Czech Republic; 5 Institute of Genetics and Animal Biotechnology of the Polish Academy of Sciences, 05-552 Magdalenka, Poland; atanas.atanasov@univie.ac.at; 6 Institute of Neurobiology, Bulgarian Academy of Sciences, 1113 Sofia, Bulgaria; 7 Department of Pharmaceutical Sciences, University of Vienna, 1010 Vienna, Austria; 8 Ludwig Boltzmann Institute for Digital Health and Patient Safety, Medical University of Vienna, 1090 Vienna, Austria; 9 Regional Institute of Gastroenterology and Hepatology, 400000 Cluj-Napoca, Romania; na_hajjar@yahoo.com

**Keywords:** non-small-cell lung cancer, small-cell lung cancer, targeted sequencing, patients

## Abstract

Background: Lung cancer remains one of the most diagnosed malignancies, being the second most diagnosed cancer, while still being the leading cause of cancer-related deaths. Late diagnosis remains a problem, alongside the high mutational burden encountered in lung cancer. Methods: We assessed the genetic profile of cancer genes in lung cancer using The Cancer Genome Atlas (TCGA) datasets for mutations and validated the results in a separate cohort of 32 lung cancer patients using tumor tissue and whole blood samples for next-generation sequencing (NGS) experiments. Another separate cohort of 32 patients was analyzed to validate some of the molecular alterations depicted in the NGS experiment. Results: In the TCGA analysis, we identified the most commonly mutated genes in each lung cancer dataset, with differences among the three histotypes analyzed. NGS analysis revealed *TP53*, *CSF1R*, *PIK3CA*, *FLT3*, *ERBB4,* and *KDR* as being the genes most frequently mutated. We validated the c.1621A>C mutation in *KIT*. The correlation analysis indicated negative correlation between adenocarcinoma and altered *PIK3CA* (r = −0.50918; *p* = 0.0029). TCGA survival analysis indicated that *NRAS* and *IDH2* (LUAD), *STK11* and *TP53* (LUSC), and *T53* (SCLC) alterations are correlated with the survival of patients. Conclusions: The study revealed differences in the mutational landscape of lung cancer histotypes.

## 1. Introduction

GLOBOCAN 2020 indicates lung cancer as being the second most diagnosed cancer after breast cancer, while still being the leading cause of cancer-related deaths [1], despite the decline recorded in the last decade regarding lung cancer incidence and mortality rates. Based on the histological aspect, lung cancers can be divided into two main types—non-small-cell lung cancer (NSCLC) and small-cell lung cancer (SCLC)—the first being responsible for more than 80% of lung cancers, while the latter accounts for about 15% of bronchogenic carcinomas. High mortality rates in lung cancer are not type-specific, with the survival rate being directly linked to the stage at diagnosis. More precisely, the 5-year survival rate is about 14% in stage III and 1% in stage IV NSCLC, and 8% in stage III and 2% in stage IV SCLC [2]. Still, the late diagnosis is not the only cause for high mortality in lung cancer. Another cause is the fact that lung cancer treatments appear to be less effective than treatments for other types of cancer (i.e., breast cancer) [3]. Nonetheless, the mutational burden of lung tumors appears to be the most critical aspect that influences response to therapy and, thus, survival rates of lung cancer patients. One important element to consider in lung cancer is the higher mutational burden of the patients with a smoking history compared to the age-related lung cancer patients [4]. Regarding the somatic mutations identified in lung cancer tumors, it is well known that the number of driver mutations in cancer is correlated to the number of somatic mutations. Therefore, an increase in the number of somatic mutations would be succeeded by an increase in the driver mutations number. In this context, the high mutational burden of lung cancer tumors hinders the attempts to identify a single driver mutation that can be druggable for therapeutic purposes. Therefore, most cancer patients display a high mutational burden that appears during a process of accumulation of driver mutations that confer to the tumor cell a selective advantage. This way, the targeted therapies available in clinical practice for oncologists to treat lung cancer are restricted to a few options [2].

However, the generous amounts of data regarding genetic alterations (including mutation detection and copy number alterations) that are now available using NGS technology favor the detection of new molecular biomarkers for early detection and new targetable molecules in precision medicine for a personalized treatment that would improve the lung cancer patient’s outcome. The impact of the NGS technologies is remarkable as it offers data support to understand the biological processes underlying oncogenesis and favors personalizing patient care in clinical practice. Nowadays, using NGS, the research community is making substantial progress in detecting new causative mutations in lung cancer tumors, to identify new biomarkers to favor early diagnosis, and these elements guide clinicians in selecting a properly targeted therapy for lung cancer patients based on their molecular profile [5,6,7,8].

**Figure 1:**
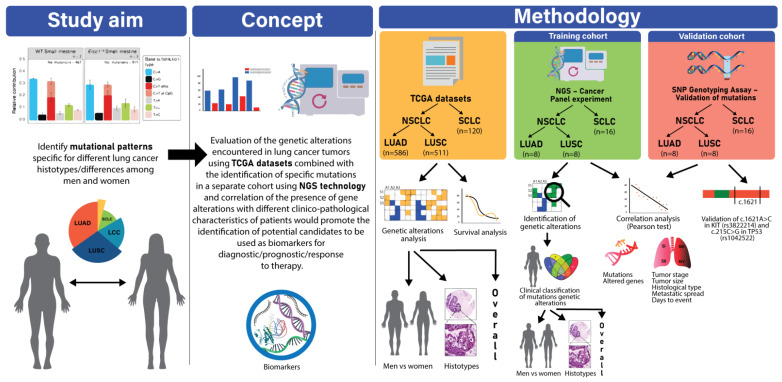
Flowchart of the study design.

This study hypothesized that the molecular alterations in lung cancer patients are associated with the morphological/histological classification of this pathology. We hypothesized that each histological type is characterized by a set of specific mutations/alterations in the cancer genes panel used for the targeted sequencing experiment and that we might be able to discriminate between histological types based on the mutational profile of each histotype. We aimed to characterize the genetic alterations encountered in patients included in the datasets for lung cancer (LUAD, LUSC, and SCLC) in the TCGA database and to unravel specific altered pathways for each histotype. Another objective was to assess the mutational profile of lung cancer patients using a 46-gene panel in a targeted sequencing experiment and to identify commonly mutated genes in patients with the same lung cancer subtype. Another focus of this study was to identify correlations between genetic alterations and clinicopathological characteristics of the patients in the study (Figure 1).

## 2. Materials and Methods

### 2.1. TCGA Datasets and Statistical Analysis

For the TCGA (The Cancer Genome Atlas) data, analysis began with the download and visualization of the available data from the CBioPortal webpage (v 4.0.1). The datasets downloaded included the most comprehensive datasets for the two subtypes of lung cancer (NSCLC and SCLC) in three separate datasets for lung adenocarcinoma (LUAD) (586 tumor samples), lung squamous cell carcinoma (LUSC) (511 tumor samples), and small-cell lung cancer (SCLC) (120 tumor samples).

The statistical analyses were performed using GraphPad Prism v.6.0. The Pearson test was used for determining statistical associations between most altered genes identified as carrying also pathogenic mutations; altered genes and tumor stage; tumor size; histological type; lymph node involvement; metastatic spread; days to event (death).

### 2.2. Patient Cohorts

For this study, the first cohort of patients consisted of a total of 32, of which 16 samples were non-small-cell lung carcinoma (adenocarcinoma and squamous cell carcinoma) and 16 were small-cell lung carcinoma. In this cohort, we used primary lung tumor biopsies and whole blood samples from 25 males and 7 females. The sampling was performed from patients undergoing biopsies for diagnostic (tumor and adjacent normal tissue) for stage III and IV pulmonary cancer. The patients were enrolled in the study between December 2016 and January 2018. The second cohort also included 32 patients—16 NSCLC and 16 SCLC (9 females and 23 males)—diagnosed with pulmonary cancer between February 2018 and May 2019. All patients were provided with detailed information regarding the present study and gave their informed consent. This research was approved by the Ethical Committee of Leon Daniello Pulmonology Hospital, Cluj-Napoca, Romania no. 264/26.06.2018 and the Ethical Committee of Iuliu Hatieganu University of Medicine and Pharmacy no. 438/24.11.2016. The clinicopathological characteristics of these patients are listed in Table 1 and the individual characteristics in Table 2.

### 2.3. Sample Processing and DNA Extraction

Lung tissue biopsy samples obtained from the patients described in the previous section were processed following the steps required using the Trizol Reagent (Ambion) according to the manufacturer’s instruction for DNA extraction. Therefore, after the suspension of the tissue sample in the Trizol reagent, the sample was snap-frozen in a liquid nitrogen bath in order to proceed with the homogenization step using a polytron. The following steps included the addition of 160 μL of chloroform. The separation of the aqueous phase for the DNA precipitation was performed in the tube using pure ethanol. After the two washing steps with 75% ethanol, DNA is resuspended using 40 μL of nuclease-free water. DNA extraction from 200 µL whole blood matched samples was performed using the PureLink™ Genomic DNA Mini Kit (Thermo Fisher Scientific, Waltham, MA, USA) according to the manufacturer’s instructions. The concentration and quality of the gDNA were assessed using a NanoDrop-1000 spectrophotometer.

### 2.4. Sequencing Protocol and Data Analysis

The targeted sequencing protocol was performed using the Ion Ampliseq Cancer Panel. This panel includes primer pairs for 190 amplicons in 46 genes with known involvement in cancer (ABL1, AKT1, APC, ALK, ATM, BRAF, CDH1, CDKN2A, CSF1R, CTNNB1, EGFR, ERBB2, ERBB4, FBXW7, FGFR1, FGFR2, FGFR3, FLT3, GNAS, HNF1A, HRAS, IDH1, JAK2, JAK3, KDR, KIT, KRAS, MET, MPL, NPM1, NOTCH1, NRAS, PIK3CA, PDGFRA, PTEN, PTPN11, RET, RB1, SMAD4, STK11, SRC, SMARCB1, SMO, TP53, and VHL1). The panel covers 739 COSMIC mutations found in 604 loci having coverage of 97%. For the library preparation, we used the Ion Ampliseq™ Library Kit 2.0 (Thermo Fisher Scientific, Waltham, MA, USA, which required 20 ng of gDNA. The purification step was performed using AMpure XP Beads (Beckman Coulter), and the purified libraries were assessed for quantification with the Qubit HS DNA kit. Ion 316 Chip (Thermo Fischer Scientific) with four barcoded 100 pm diluted libraries for the upload of the samples in the Ion Torren PGM Machine (Thermo Fischer Scientific, Waltham, MA, USA). The sequencing protocol was employed with the Ion PGM Sequencing 200 kit according to the manufacturer’s instructions.

Signal processing, base calling, and alignment to the *hg19* reference genome operated on the Torrent Suite 5.6 (Life Technologies). The reference genome was used for the comparison and it represents the normal sequence, as previously described [9,10,11]. For the downstream analyses of variant calling and data trimming alignment, the Ion Reporter 5.6 software was used. The same software was used for annotations; we performed the transfer of the VCF files for each sample using the following parameters: coverage ≥ 100 for tissue samples and ≥50 for blood samples and *p*-value ≤ 0.05. For the clinical assessment of the mutations (benign/likely benign/neutral/likely pathogenic/pathogenic), the COSMIC (Catalogue of Somatic Mutations in Cancer) (https://cancer.sanger.ac.uk/cosmic (accessed on 12 November 2021)) and the ClinVar databases were interrogated (https://www.ncbi.nlm.nih.gov/clinvar/ (accessed on 12 November 2021)).

### 2.5. Mutation Validation—SNP Genotyping Assay

Using TaqMan™ SNP Genotyping Assay for c.1621A>C in *KIT* (rs3822214) and c.215C>G in *TP53* (rs1042522) mutations, we experimented validating the two mutations following the next protocol: after the preparation of the 20x assay dilutions, the PCR mix consisted of 2.5 μL of TaqMan MasterMix, 0.25 μL of the assay (rs1042522/rs3822214) and 1 μL of DNA (20 ng/μL) and 1.25 μL of ultrapure H_2_O. The PCR program for the genotyping assay consists of the following incubation steps: 95 °C for 10 min, followed by 40 cycles of 95 °C for 15 s and 60 °C for 1 min. For this experiment, all samples were analyzed in duplicate. All the reactions were performed on a quantitative real-time PCR machine ViiA7 (Thermo Fisher Scientific, Waltham, MA, USA). Additionally, the analysis was performed with the PCR instrument incorporated software.

## 3. Results

### 3.1. Genetic Alterations in the TCGA Datasets

The TCGA data analysis began with downloading the mutation and can file for each dataset of lung cancer type: LUAD, LUSC, and SCLC. After selecting the top 30 genes with frequent alterations (with known status of cancer gene), we combined the results obtained for each dataset and the removal of the duplicate retrieved 54 genes that were selected as the most mutated cancer genes in lung cancer TCGA datasets and were used for the analysis of the genetic alterations.

**Figure 2:**
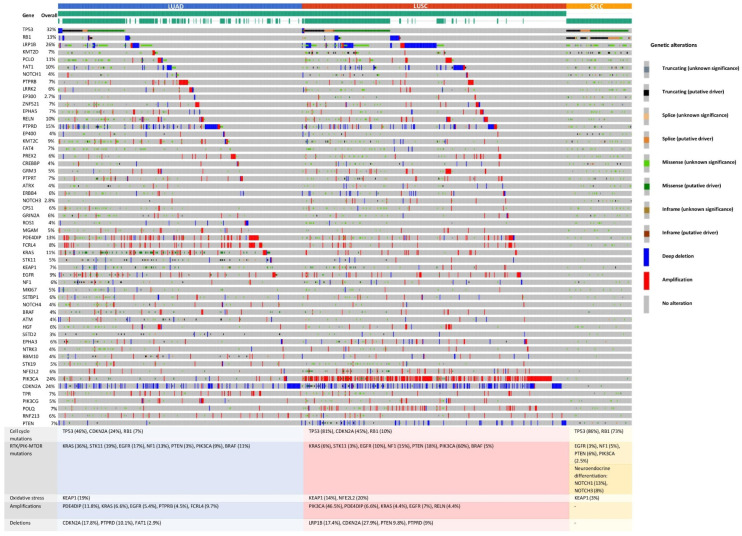
Genetic alterations identified in the TCGA lung cancer datasets. (top) Lung adenocarcinoma (LUAD) (blue), lung squamous cell carcinoma (LUSC) (red), and small-cell carcinoma (SCLC) (orange). Overall genetic alterations in each of the three main histotypes of lung cancer in the top 54 most common genes that harbor gene alterations (left). Review of the prevalence of different genetic alterations in key molecular pathways separated based on the histological type (bottom).

As expected, TP53 was the gene with the most frequent alterations across the three datasets. TP53 had an overall frequency of 32% in the samples included in all three datasets analyzed. In LUAD, TP53 frequency was 46%; in LUSC, it was 81%; and in SCLC, it was 86%. A high frequency of alterations was depicted in LRP1B (26%), PIK3CA (24%), CDKN2A (24%), PTPRD (15%), RB1 (13%), PDE4DIP (13%), PCLO (11%), KRAS (11%), FAT1 (10%), and RELN (10%). The separate analysis revealed that in the LUAD dataset, the most altered genes were KRAS (36%), CDKN2A (24%), KEAP (19%), STK11 (19%), EGFR (17%), NF1 (13%), and BRAF (11%). The analysis on the LUSC dataset indicated the PIK3CA (60%), CDKN2A (45%), PTEN (18%), NF1 (15%), and EGFR (10%) genes as the most frequently mutated genes. When the SCLC dataset was assessed, RB1 (73%) and NOTCH1 (13%) were the most altered cancer genes. All these genes are part of processes involved in oncogenesis, such as cell cycle regulation (TP53, RB1, and CDKN2A), RTK/PIK–MTOR pathway (KRAS, STK11, EGFR, BRAF, NF1, PTEN, PIK3CA), oxidative stress (KEAP1, NFE2L2), and neuroendocrine differentiation (NOTCH1) (Figure 2).

The second analysis using the TCGA datasets was CNA for LUAD and LUSC (as it was not available for SCLC). In LUAD, FCRL4 (9.7%), KRAS (6.6%), EGFR (5.4%), and PTPRB (4.5%) were the identified genes with copy number gain, while CDKN2A (17.8%), PTPRD (10.1%), and FAT1 (2.9%) were distinguished as genes with copy number loss. In the second dataset, LUSC, PIK3CA (46.5%), EGFR (7%), PDE4DIP (6.6%), KRAS (4.4%), and RELN (4.4%) were labeled with copy number gain, while CDKN2A (27.9%), LRP1B (17.4%), PTEN (9.8%), and PTPRD (9%) appeared to be the cancer genes with copy number loss (Figure 2).

Using the TCGA datasets for lung cancer patients, we also took a look at the genetic alterations identified in each dataset separating the frequencies for men and women.

**Figure 3:**
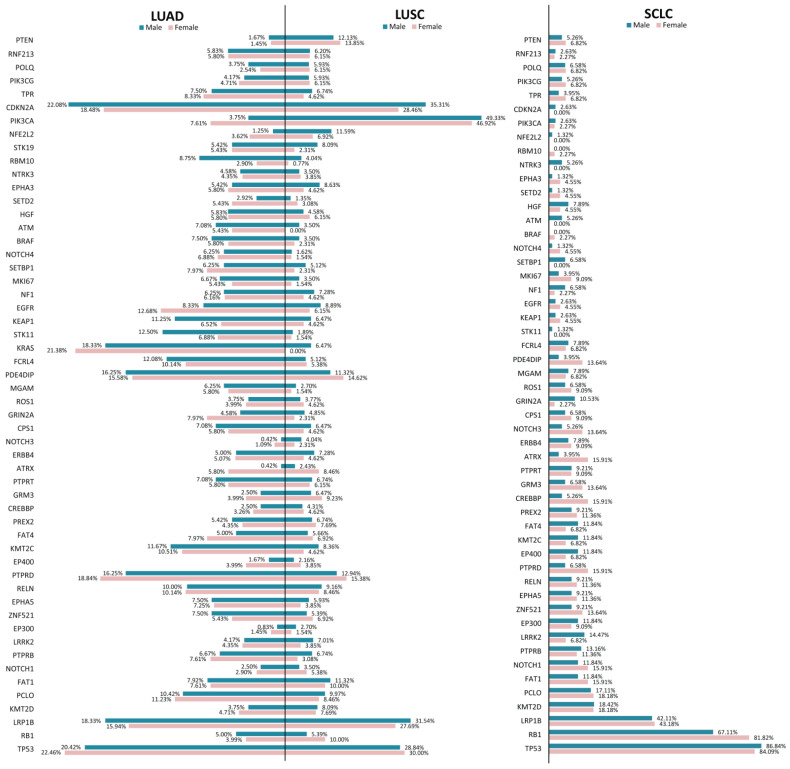
Bar graphs represent the frequencies of altered genes in women (pink) and men (blue) for each dataset (LUAD, LUSC, and SCLC).

As illustrated in Figure 3, in LUAD patients, TP53 was altered in 22.46% of women and 20.42% of men, while in LUSC, TP53 was altered in 30% of women and 28.84% of men. The highest frequency of TP53 alterations was encountered in women (84.09%) and men (86.84%) in the SCLC dataset. This is also the case for RB1, which was altered in 81.82% of women and 67.11% of men in SCLC, while in LUAD and LUSC, the frequencies were less than 10% of the men/women. LRP1B was encountered altered in 18.33% of men and 15.94% of women in LUAD, and31.54% of men and 27.69% of women in LUSC; in SCLC, the alteration frequencies in both men and women were about 40%. The clearest differences in terms of alteration frequencies in men and women were observed in the fact that in LUAD, ATRX, KRAS, and EGFR appeared to be altered with higher frequency in women (5.8%, 21.38%, and 12.68%, respectively), than in men (0.42%, 18.33%, and 8.33%, respectively). In LUSC, visible differences between men and women were observed in ATRX (alterations being more frequent in women—8.46% compared to 2.43% in men) and CDKN2A (alterations being more frequent in men—35.31% compared to 28.46% in women). In SCLC, it can be observed that cancer gene alterations appear to be more frequent in women, the most consistent differences being observed in PTRD, CREBBP, GRM3, ATRX, NOTCH3, and PDE4DIP, while ATM appears to be altered in 5.26% of men, and no alterations were depicted in women.

### 3.2. Clinicopathological Characteristics of the Cohort

**Figure 4:**
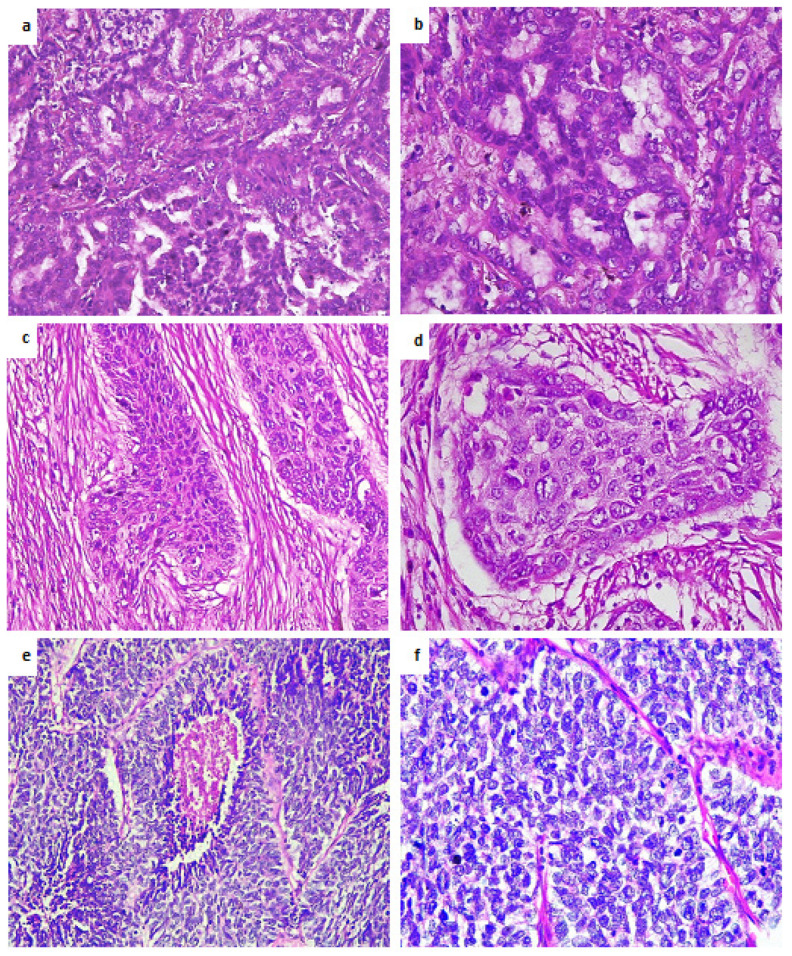
Hematoxylin–eosin staining of lung tumor tissue samples: (**a**) Lung adenocarcinoma at 200× magnification, showing classical glandular architecture; (**b**) Lung adenocarcinoma at 400×, nuclear atypia, nuclear polymorphism and cytoplasmatic secretion vesicle; (**c**) Lung squamous cell carcinoma at 200×, presenting islands of large polygonal cancer cells disposed of in a fibrous stroma; (**d**) High-magnification of a lung squamous cell carcinoma, 400×, showing the morphological details of the malignant cells with large adherent and polygonal cells, atypical and polymorph nuclei and presence of mitoses; (**e**) Small-cell lung carcinoma at 200× magnification showing a hypercellular tumor consisting of cells with round, oval nuclei, and scant cytoplasm, ill-defined cellular borders with a central zone of necrosis. (**f**) SCLC at 400× magnification highlights characteristic nuclear features such as nuclear molding, finely dispersed chromatin pattern, absence of nucleoli and presence of multiple mitoses.

The main characteristics of the patients included in the study cohort are listed in Table 1. The patients included in the sequencing experiment were between 53 and 81 years, with a mean age of 62 years. Males represented a majority with 25 cases (78.2%), while women represented a minority with 7 cases (21.8%). The cohort of 32 patients used in the mutation validation experiment were between 42 and 81 years, with a mean age of 62 years. Males represented approximately 72% (23 cases), while women represented about 28% (9 cases) of the total. Each cohort included 16 NSCLC samples (8 lung adenocarcinomas and 8 squamous cell carcinomas of the lung) and 16 SCLC samples. For this study, we only selected patients with stage III and IV lung cancer at diagnosis. The pTNM classification is detailed in Table 1. Table 2 details the individual clinicopathological characteristics for the patients included in both training and experimental cohorts. The visualization of lung tumor tissue samples (NSCLC and SCLC) stained with hematoxylin–eosin is represented in Figure 4.

### 3.3. Mutations Identified in the Experimental Cohort

When we sequence a patient’s genome and compare it to the reference genome, the reference represents the normal sequence. Therefore, any identified variation could be considered responsible for the malignant phenotype of lung cancer patients. After establishing the parameters for filtering the significant mutations in the targeted sequencing experiment (*p* > 0.05, coverage > 100× or >50×), we proceeded to the filtering out of the synonymous mutations, as they do not affect the phenotype of the transformed cell. The selected mutations were classified as benign/likely benign/neutral/likely pathogenic/pathogenic according to the FATHMM score and according to the ClinVar interpretation. Moreover, according to The International HapMap Consortium, minor allele frequency (MAF) is widely used in population genetics studies because it provides information to differentiate between common and rare variants in the population); a variant is considered rare if the MAF ≤ 0.05, and low if MAFs <  0.1 [12,13]. Table 3 lists all the pathogenic mutations detected after filtering out the 32 tumor biopsy samples and matched whole blood samples. As can be observed in Supplementary File S1, the majority of the pathogenic alterations that were further analyzed in our study have a MAF of 0 and very few a MAF of 0.1, indicating that these alterations might be linked to lung cancer pathogenesis (Supplementary File S1).

**Figure 5:**
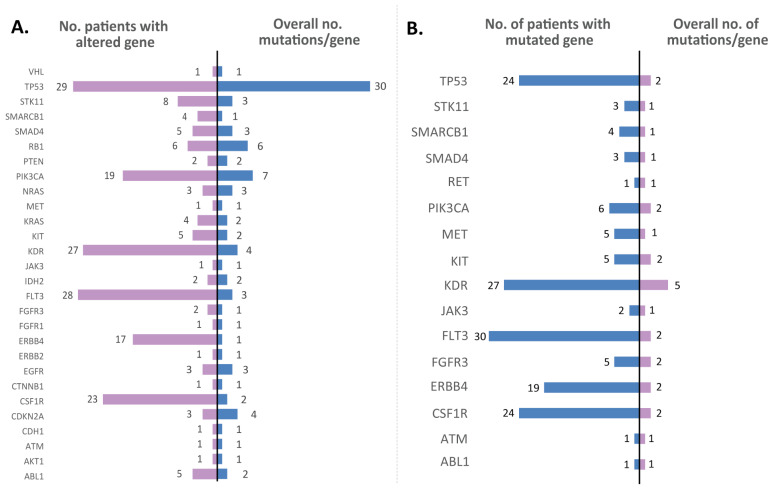
Bar graph representing the number of different mutations identified in each gene (left) and the number of patients in which at least one variant was detected for each gene (right) in tumor tissue samples (**A**) and whole blood samples (**B**).

Figure 5 illustrates all the altered genes in the analysis, alongside the number of different mutations encountered in each gene and the number of patients harboring at least one mutation in the gene for both tumor biopsy and whole blood samples. According to the data generated in the sequencing experiment, the most altered genes in the tumor tissue samples were TP53, CSF1R, PIK3CA, FLT3, and KDR, while in the matched blood samples, KDR was encountered with frequent alterations. As illustrated, CSF1R (72%), ERBB4 (53%), FLT3 (87%), KDR (84%), PIK3CA (59%), and TP53 (90%) appeared to be altered in a higher number of tumor tissue biopsy samples, while frequently altered genes in blood samples were FLT3 (93.7%), KDR (84.3%), CSF1R (75%), TP53 (75%), and ERBB4 (59.3%). Regarding c.*37delA and c.*35insT, alterations in the CSF1R were detected in the majority of the samples analyzed, their categorization is still unknown, but is most likely benign. Pathogenic mutations were detected in the following genes: ABL1, CDKN2A, CTNNB1, ERBB2, IDH2, KIT, NRAS, PIK3CA, PTEN, RB1, RET, SMAD4, STK11, and TP53. A particular case is the c.215C>G (p.Pro72Arg) mutation in TP53. This alteration was found in 65% of the tumor tissue samples and 75% of the matched blood samples from patients in the cohort and is associated with drug response.

As they were detected in a higher number of patients (c.215C>G in TP53 (rs1042522), c.1621A>C in KIT (rs3822214), and c.3196G>A in PIK3CA), these alterations were taken into consideration for subsequent studies, their classification being either pathogenic or being associated with drug response. Moreover, there are many genes with only one detected mutation that appears to be present in only one patient. Given the high mutational burden of the patients in the experimental cohort and the reduced size of the cohort analyzed, further studies are required to establish a common mutational pattern for each histological type of lung cancer.

**Figure 6:**
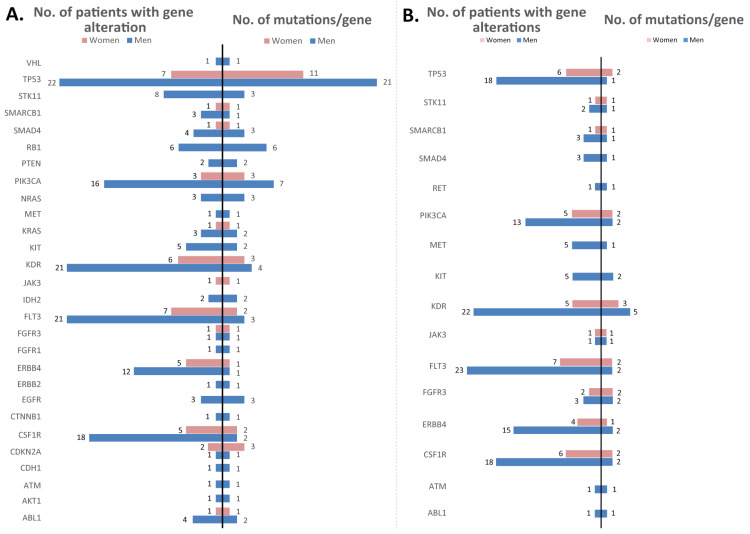
Bar graph representing the number of different mutations identified in each gene (left) and the number of patients in which at least one variant was detected for each gene (eight) in women (pink) and men (blue) in tumor tissue samples (**A**) and whole blood samples (**B**).

**Figure 7:**
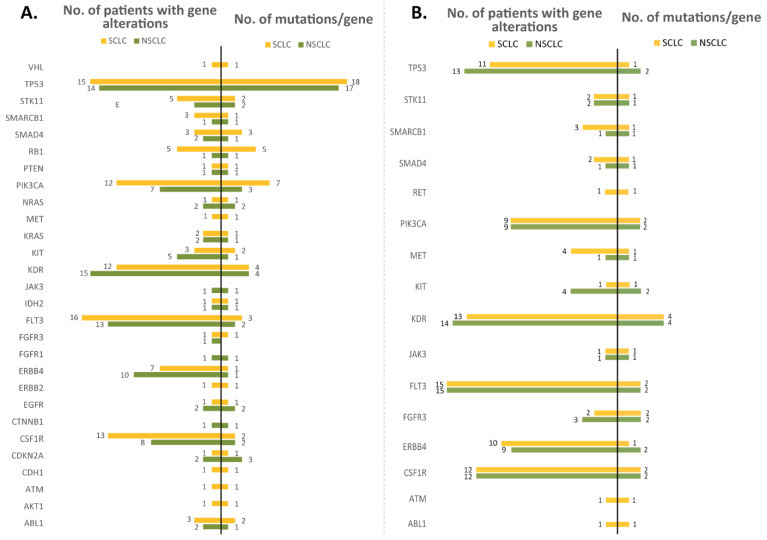
Bar graph representing the number of different mutations identified in each gene (right) and the number of patients in which at least one variant was detected for each gene (left) in NSCLC samples (green) and SCLC samples (yellow) in tumor tissue samples (**A**) and whole blood samples (**B**).

When assessing the number of different mutations identified and the number of patients with altered genes separately on men and women, it was revealed that in women, the number of mutated genes appeared to be lower than in men. As expected, in the tumor tissue samples, TP53 was the gene with frequent alterations in both men and women, followed by KDR, FLT3, PIK3CA, ERBB4, and CSF1R. One observation is that KIT, STK11, and RB1 were found mutated only in men, and alterations in these genes were encountered in a large number of patients. In the matched blood samples, KDR was encountered with frequent alterations, followed by CSF1R, FLT3, FGFR3, and PIK3CA, in both men and women. Moreover, SMAD4, MET, and KIT were identified as altered only in blood samples from men (Figure 6). Nevertheless, these observations need further studies for statistical validation regarding the differences in the mutational profiles of men vs. women. When the mutation analysis was performed separately on NSCLC and SCLC samples to compare the similarities/differences, similarities were observed in the high number of alterations depicted in TP53, PIK3CA, and KDR; and in the high number of patients harboring mutations in TP53, PIK3CA, KDR, FLT3, and CSF1R in tumor samples. In the blood samples, KDR had a higher number of alterations and FLT3, KDR, CSF1R, TP53, ERBB4, and PIK3CA were altered in a higher number of patients. When assessing the differences, it was observed that in SCLC patients, RB1 was depicted with a higher number of different mutations and this gene was altered in a higher number of patients (in tumors), while in blood samples, RET, ATM, and ABL1 appeared altered only in the SCLC group. Moreover, in the SCLC group, the patients appeared to have more different genes mutated than in the NSCLC group (Figure 7).

### 3.4. Mutation Validation

Two of the variants that were detected in several patients with lung cancer were tested for further validation, as they were classified as pathogenic or associated with drug response according to ClinVar. In this respect, we used the TaqMan SNP Genotyping assay for the following gene coding variants: for c.1621A>C in KIT (rs3822214) and c.215C>G in TP53 (rs1042522). This experiment properly validated the c.1621A>C in KIT (rs3822214) assay, as the proportion of patients (6 out of 31) with a positive identification was similar to the proportion in the sequencing experiment. The poor DNA quality did not allow the identification of a result for one sample. The assay for TP53 (rs1042522) was not properly validated, as the proportion of patients (3 out of 27) with a positive identification (11%) was different from the proportion in the sequencing experiment (21 out of 32) (65%). Poor DNA quality in five samples hampered the possibility of identifying a result.

### 3.5. Statistical Analysis

Overall patient survival was assessed for the three TCGA datasets (LUAD, LUSC, SCLC) for the 13 genes identified as harboring pathogenic mutations in the sequencing experiment. The survival analysis revealed that in the TCGA LUAD datasets, the presence of NRAS, IDH2, and CDKN2A alterations is correlated with the survival of patients, the latter gene reaching a borderline statistical significance. For LUSC datasets, TP53, STK11, and CDKN2A alterations appear to be correlated with the survival of patients, the latter gene reaching a borderline statistical significance. In the SCLC dataset, only STK11 appeared to reach statistical significance in the survival analysis (Table 4). These results suggest that overall survival of patients with the above mentioned altered genes for each dataset is reduced compared to the unaltered group.

Pearson correlation analysis is represented in Table 5. For this analysis, we selected the genes altered in a higher number of patients with at least one pathogenic mutation to assess the correlation with the clinicopathological information of the patients (tumor stage, tumor size, histological type, lymph node involvement, metastatic spread, days to event (death)). For the correlation analysis, we selected the genes based on the pathogenicity score of the alterations obtained by interrogating the two mentioned mutation databases (ClinVar and COSMIC). This parameter was chosen given its essential role in evaluating lung cancer biology. Survival data (days to event) were available for 26 out of 32 patients from the training cohort and for 30 out of 32 from the validation cohort. Significant values for different correlations are bolded in Table 5. As expected, the presence of metastases was correlated with stage (r = 0.96862; *p* = 0.000). A strong negative correlation was identified between adenocarcinoma and altered PIK3CA (r = −0.50918; *p* = 0.0029). Regarding the smoking status, the analysis revealed a moderate positive correlation between former smokers and altered PIK3CA and (r = 0.364698; *p* = 0.0401), and a moderate negative correlation between never smokers and altered PIK3CA (r = −0.3647; *p* = 0.0401); a moderate negative correlation between former smokers and adenocarcinoma (r = −0.30949; *p* = 0.013); and a moderate positive correlation between never smokers and adenocarcinoma (r = 0.309492; *p* = 0.013) (Table 5).

## 4. Discussion

In this study, we aimed to identify the genetic alterations in cancer genes using three different lung cancer datasets (LUAD, LUSC, and SCLC), to identify common mutational patterns specific for each histotype, to address the differences between men and women, and to compare the results in the TCGA datasets with the genetic alterations observed in an NGS experiment performed on a cohort of 32 lung cancer patients. Another focus of this paper was to assess the impact of pathological alterations on the clinical outcome of the patients in our cohorts and whether these alterations can be correlated with different clinicopathological characteristics, such as histological type, stage, lymph node involvement, metastasis, smoking status, etc.

In lung cancer studies, implementing NGS approaches using brain metastases samples from patients with metastatic NSCLC reported *TP53* as being the most frequently altered gene in the samples, followed by *KRAS*, *FGFR3*, *CDKN2A,* and *VHL.* The authors also identified various targetable mutations that may be used for targeted approaches to improve patients’ outcomes. Still, the high mutational burden reported in this study questions the ease of applying targeted therapies with high efficiency in metastatic NSCLC [14].

In the three TCGA lung cancer datasets (LUAD, LUSC, SCLC), *TP53* was the most frequently (32%) mutated gene, with differences among the three cohorts: 46% in LUAD, 81% in LUSC, and 86% in SCLC. The following most altered cancer genes were *LRP1B* (26%), *PIK3CA* (24%), *CDKN2A* (24%), *PTPRD* (15%), *RB1* (13%), *PDE4DIP* (13%), *PCLO* (11%), *KRAS* (11%), *FAT1* (10%), and *RELN* (10%), and these results partially overlap with the most altered genes depicted in the experimental cohort. Additionally, in these datasets, we depicted differences in the molecular alterations specific for each histotype: in LUAD, the most altered genes (except *TP53*) were *KRAS* (36%), *CDKN2A* (24%), *KEAP* (19%), *STK11* (19%), *EGFR* (17%), *NF1* (13%), and *BRAF* (11%); in the LUSC dataset, the most altered genes were *PIK3CA* (60%), *CDKN2A* (45%), *PTEN* (18%), *NF1* (15%), and *EGFR* (10%); in SCLC, *RB1* (73%) and *NOTCH1* (13%) were the most mutated cancer genes (Figure 2). In these datasets, we were able to identify patterns of mutated genes specific for the three histological types. Unlike the cohort in the targeted sequencing experiment where we used comparable numbers of patients for each class of lung cancer (16 NSCLC and 16 SCLC specimens), in the TCGA cohorts, SCLC sample data were available for only 120 patients, while the NSCLC group included 1097 sample data (586 LUAD and 511 LUSC), thus making this analysis more characteristic for the NSCLC group, hence the differences in the TCGA datasets versus the experimental cohort in terms of characteristic mutations identified.

When we assessed the genetic alterations identified in each lung cancer TCGA dataset separating the cancer genes’ alteration frequencies observed in men and women, we identified several genes with visible differences in terms of genetic alterations between men and women. One notable difference was observed in *RB1*, which was altered in 81.82% of women and 67.11% of men in SCLC, while in LUAD and LUSC, the frequencies were less than 10% of the men/women. We also identified that *ATRX*, *KRAS,* and *EGFR* appeared to be altered with higher frequency in women (5.8%, 21.38%, and 12.68%, respectively), than in men (0.42%, 18.33%, and 8.33%, respectively) in the LUAD datasets. In LUSC, visible differences between men and women were observed in *ATRX* (women—8.46%, compared to men—2.43%) and *CDKN2A* (men—35.31%, compared to women—28.46%). We also observed that in the SCLC dataset, cancer gene alterations appeared to be more frequent in women, with one exception—*ATM*, which appears to be altered only in men (Figure 3).

In our experiments, *TP53* appeared as the most frequently altered gene in the tissue samples analyzed (more than 90%), while in blood samples, *FLT3* was the most frequently altered gene (more than 90%). *FLT3* harbors germline mutations that appear to be present in the majority of lung cancer patients analyzed in this study. According to the lung cancer literature, SCLC specimens encounter the highest frequency of *TP53* alterations [15,16]. In the samples analyzed in our study, SCLC samples were identified as harboring *TP53* alterations (more than 93% of the samples) with a frequency that is slightly higher than in the NSCLC specimens analyzed (87% of the samples) in the tumor tissue, but this tendency was not observed in the blood samples, *TP53* being altered in 68% of SCLC and 81% of NSCLC samples (Figure 7). The majority of these mutations were catalogued as pathogenic, while one mutation was associated with drug response (c.215C>G) (Table 3). This variation in *TP53* was encountered in more than 65% of the tumor tissue samples and was selected for validation using the SNP genotyping assay in a second cohort of matched lung cancer patients, but was not properly validated in the second cohort, as it was detected in only 11% of the analyzed samples. Frequent abnormalities of *TP53* in lung cancer patients are associated with worse overall survival and resistance to therapy [17,18,19].

Other frequently mutated genes in the tumor tissue samples used in our lung cancer study were *FLT3* (altered in 87% of the samples), *KDR* (84%), *CSF1R* (72%), *PIK3CA* (59%), and *ERBB4* (53%). Similar proportions were also observed in the mutational profile of the blood samples: *FLT3* (altered in 93% of the samples), *KDR* (84%), *CSF1R* (75%), and *ERBB4* (59%). The variations identified in *CSF1R* were not classified in the COSMIC or ClinVar databases, but as identified in most of the samples analyzed (both tissue and blood), are considered germline mutations and appear to be most likely benign. *FLT3* alterations were studied mostly in acute myeloid leukemia, and are particularly involved in cell differentiation, survival, and proliferation, in a mechanism involving PI3K, RAS, and STAT5 [20]. Moreover, *FLT3* is a druggable target, as first- and next-generation inhibitors were already developed for precise treatments and in order to overcome acquired resistance [21,22,23]. In our study, two of the *FLT3* variations detected are known to be neutral, and one has no classification yet. *KDR* is a tyrosine kinase gene that may function as a proto-oncogene, as mutations in *KDR* appear to modulate angiogenesis, being involved in cell proliferation, migration, and survival. While most of the identified mutations appear to be neutral, the others are not classified yet; in a previous study involving c.1416A>T (p.Gln472His) variation with a frequency of 33% in patients, it was suggested as functioning as a proto-oncogene in LUAD [24]. In our cohort, this mutation appeared as germline, having a frequency of about 47% of the patients (in both tissue and blood samples), yet the FATHMM score indicates it as neutral, while it was not classified yet in the ClinVar mutation databases. *PIK3CA* was identified in this cohort as harboring seven different mutations, most of them being labeled as pathogenic. In total, at least one *PIK3CA* mutation was identified in 19 tumor samples from patients (59% frequency), and 7 tumor samples from patients (20%) had at least one pathogenic mutation in *PIK3CA*. Other studies reported *PIK3CA* as harboring pathogenic mutations in 4% of the cohort of 186 NSCLC patients [25]. *ERBB4* appeared to be altered in 17 tumor samples (53%) and 8 blood samples (25%), but this gene harbored one mutation classified as neutral and another one with no classification (Figure 5). Our study revealed that *KIT*, *STK11*, and *RB1* were found mutated only in men’s tumor samples, while *SMAD4*, *MET*, and *KIT* were identified as altered only in blood samples from men; alterations in these genes were encountered in a large number of patients, indicating there are some differences in terms of mutations in the group of women vs. men (Figure 6). When comparing the mutations identified in the NSCLC and SCLC groups, we observed that *RB1* was depicted with a higher number of different mutations in tumor samples and this gene was altered in a higher number of patients in the SCLC group (Figure 7); this result was consistent with the TCGA data analysis (Figure 2).

**Figure 8:**
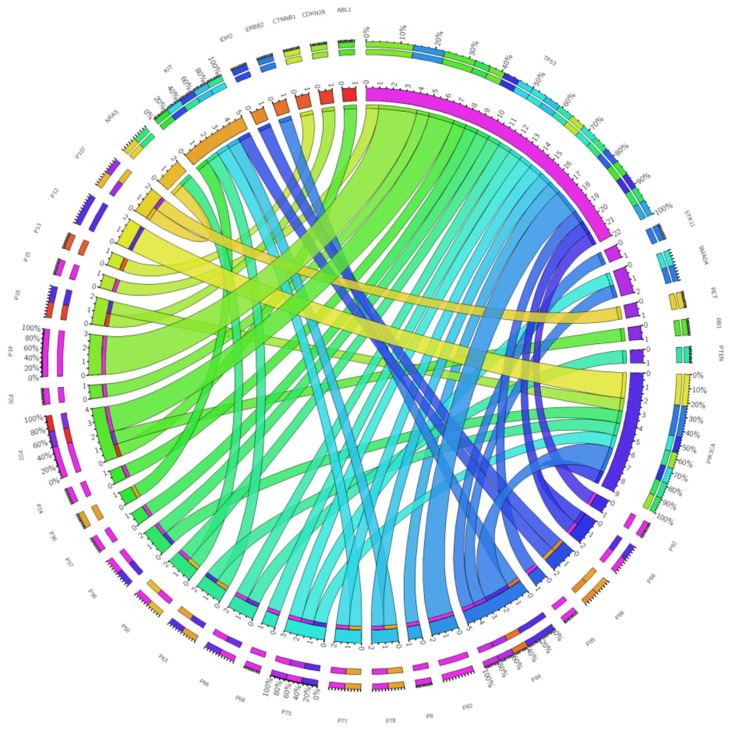
Circos representation of the number of pathogenic mutations identified in each patient for every gene with pathogenic mutations (germline and somatic).

Other genes harboring at least one pathogenic mutation were *ABL1, CDKN2A, CTNNB1*, *ERBB2*, *IDH1*, *KIT*, *NRAS*, *PTEN*, *RB1*, *RET*, *SMAD4*, and *STK11* (Figure 8). All these genes harbor one pathogenic mutation identified in only one patient, with few exceptions—*SMAD4* and *NRAS* have two pathogenic mutations, and each mutation is encountered in only one patient; *KIT* was identified with one pathogenic mutation encountered in four different patients. The correlation analysis indicated a strong negative correlation between adenocarcinoma and altered *PIK3CA*, while a moderate correlation was noticed between time (days) to event (death) and former smokers (positive correlation) and never smokers (negative correlation). As indicated, this experiment revealed multiple genes with pathogenic alterations in lung cancer (adenocarcinoma, squamous cell carcinoma, and small-cell carcinoma), suggesting a high mutational burden for this pathology. Moreover, exposure to different carcinogens (tobacco smoke, asbestos or radon exposure, radioactive ores, etc.) might cause differences in the mutational profile of these patients. Another aspect that should be taken into consideration is that some differences may be endemic for a specific population. Besides the high mutational burden encountered in different lung cancer histotypes (that is reflected also in the reduced efficiency of targeted therapies in lung cancer), other limitations include the morphological and genetic heterogeneity previously reported by other studies [26,27,28].

## 5. Conclusions

Our study revealed *TP53*, *CSF1R*, *PIK3CA*, *FLT3*, and *KDR* as genes with most variations detected in lung cancer specimens, while *CSF1R, ERBB4*, *FLT3*, *KDR*, *PIK3CA*, and *TP53* appeared to be mutated in a higher number of patients. We also managed to underline differences in the mutational profile of patients in NSCLC vs. SCLC and men vs. women. These results partially overlapped with the mutational landscape identified analyzing lung cancer TCGA datasets. Furthermore, in our cohort, the presence of *PIK3CA* alterations was correlated with adenocarcinoma. Moreover, the mutation validation properly validated only one out of two variants selected. All these results indicate that in lung cancer tumor samples, the mutational burden is high, and even if differences in the mutational landscape of patients with LUAD, LUSC, and SCLC subtypes of lung cancer were observed, a specific mutational pattern was not established for each histological type. This situation is characteristic of malignancies with a high mutation burden as a result of the accumulation of numerous driver mutations and this limits the targeted approach in clinical practice. All these results and observations should be further investigated in larger cohorts to establish accurate mutational patterns specific for each lung cancer histotype.

## Figures and Tables

**Table 1:** Baseline clinicopathological characteristics of lung cancer patients in the cohorts.

	Training Cohort	Validation Cohort
Characteristics	No. of Patients (%)	No. of Patients (%)
Total number	32	32
Age (years), median	53–81 (62)	42–81 (62)
Gender		
Male	25 (78.2)	23 (71.9)
Female	7 (21.8)	9 (28.1)
pT stage		
pT1	1 (3.1)	-
pT2	4 (12.5)	-
pT3	10 (31.2)	7 (21.8)
pT4	17 (53.1)	25 (78.2)
pN stage		
pN0	2 (6.2)	2 (6.2)
pN1	-	2 (6.2)
pN2	20 (62.5)	22 (68.7)
pN3	10 (31.2)	6 (18.7)
pM stage		
pM0	14 (43.7)	13 (40.6)
pM1	18 (56.3)	19 (59.4)
Stage		
IIIB	14 (43.8)	11 (34.4)
IIIC	1 (3.1)	2 (6.2)
IV	17 (53.1)	19 (59.4)
Histological type		
NSCLC	16 (50)	16 (50)
SCLC	16 (50)	16 (50)
Smoking status		
Active smoker	16 (50)	16 (50)
Former smoker	13 (40.6)	13 (40.6)
Never smoker	3 (9.4)	3 (9.4)

**Table 2:** Individual clinicopathological characteristics of the 64 patients included in both cohorts.

Training Cohort								
Patient	Sex	Age at Diagnosis	TNM	Stage	Histological Type	Active Smoker	Former Smoker	Days to Event
P8	F	58	T3N2M0	IIIB	adenocarcinoma	yes	yes	116
P12	M	67	T3N2M0	IIIB	SCLC	yes	yes	41
P13	M	66	T3N3M0	IIIB	adenocarcinoma	no	yes	518
P15	M	58	T4N2M0	IIIB	squamous cell carcinoma	no	yes	311
P16	F	76	T4N3M0	IIIC	squamous cell carcinoma	yes	yes	584
P19	F	67	T3N3M1	IV	adenocarcinoma	no	no	871
P20	F	81	T4N2M0	IIIB	adenocarcinoma	no	yes	32
P22	M	58	T2N2M0	IIIB	SCLC	yes	yes	261
P23	M	60	T3N2M0	IIIB	SCLC	no	yes	336
P24	M	60	T4N2M1	IV	SCLC	no	yes	111
P31	M	61	T4N2M1	IV	squamous cell carcinoma	no	yes	
P34	M	62	T3N2M1	IV	adenocarcinoma	yes	yes	131
P36	M	65	T3N2M0	IIIB	squamous cell carcinoma	no	yes	934
P37	F	63	T3N3M1	IV	SCLC	no	no	408
P38	M	77	T4N3M1	IV	SCLC	no	yes	44
P42	M	65	T4N3M1	IIIB	SCLC	yes	yes	
P50	M	62	T4N3M1	IV	squamous cell carcinoma	yes	yes	162
P63	M	66	T4N2M1	IV	adenocarcinoma	no	yes	
P66	M	62	T4N2M1	IV	SCLC	yes	yes	62
P68	F	58	T3N2M1	IV	adenocarcinoma	no	no	582
P73	M	65	T4N3M0	IIIB	SCLC	yes	yes	116
P75	M	61	T4N2M0	IIIB	SCLC	no	yes	338
P77	M	65	T3N2M1	IV	SCLC	no	yes	
P78	M	67	T4N2M0	IIIB	SCLC	no	yes	250
P80	M	67	T2N2M1	IV	squamous cell carcinoma	yes	yes	33
P82	M	58	T4N0M0	IIIB	squamous cell carcinoma	yes	yes	32
P84	M	60	T2N2M1	IV	SCLC	yes	yes	227
P85	F	53	T4N2M0	IIIB	squamous cell carcinoma	yes	yes	70
P86	M	71	T2N0M1	IV	adenocarcinoma	yes	yes	163
P88	M	59	T4N3M1	IV	SCLC	yes	yes	
P92	M	57	T1N3M1	IV	SCLC	yes	yes	
P107	M	56	T4N2M1	IV	SCLC	no	yes	71
**Validation Cohort**								
**Patient**	**Sex**	**Age at Diagnosis**	**TNM**	**Stage**	**Histological Type**	**Active Smoker**	**Former Smoker**	**Days to Event**
P110	M	55	T3N2M1	IV	squamous cell carcinoma	yes	yes	
P118	M	70	T3N2M1	IV	SCLC	no	yes	46
P120	M	71	T4N2M0	IIIB	SCLC	yes	yes	185
P122	M	58	T4N0M1	IV	adenocarcinoma	no	yes	450
P124	M	61	T4N3M1	IV	SCLC	yes	yes	47
P129	M	60	T4N2M0	IIIB	squamous cell carcinoma	yes	yes	16
P130	F	68	T3N2M1	IV	adenocarcinoma	no	yes	143
P133	F	56	T4N2M1	IV	SCLC	yes	yes	213
P136	F	67	T3N2M0	IIIB	squamous cell carcinoma	no	yes	73
P137	F	50	T4N2M0	IIIB	adenocarcinoma	no	yes	286
P138	M	62	T4N1M1	IV	adenocarcinoma	no	no	231
P139	M	57	T4N2M0	IIIB	SCLC	no	yes	489
P140	M	69	T4N2M1	IV	SCLC	no	yes	3
P144	M	75	T4N3M1	IV	SCLC	no	yes	622
P149	M	81	T4N2M1	IV	SCLC	yes	yes	816
P150	M	60	T3N1M1	IV	SCLC	no	yes	304
P153	M	61	T4N3M0	IIIC	adenocarcinoma	no	no	
P159	M	62	T4N2M0	IIIB	squamous cell carcinoma	yes	yes	359
P160	M	80	T4N2M1	IV	squamous cell carcinoma	yes	no	888
P161	F	58	T4N3M1	IV	SCLC	yes	yes	302
P162	F	53	T4N2M1	IV	SCLC	yes	yes	887
P163	F	68	T4N2M0	IIIB	adenocarcinoma	no	yes	884
P164	M	57	T4N2M0	IIIB	SCLC	no	yes	883
P166	M	50	T4N2M1	IV	adenocarcinoma	no	yes	65
P170	M	75	T4N3M1	IV	squamous cell carcinoma	no	no	59
P171	M	56	T4N3M0	IIIC	SCLC	yes	yes	153
P181	M	73	T4N1M1	IV	SCLC	no	yes	278
P184	M	42	T4N2M0	IIIB	SCLC	yes	yes	23
P186	F	78	T4N2M1	IV	squamous cell carcinoma	yes	yes	8
P191	M	75	T3N2M0	IIIB	adenocarcinoma	yes	yes	812
P193	M	68	T3N2M0	IIIB	squamous cell carcinoma	yes	yes	319
P194	F	64	T4N2M1	IV	SCLC	yes	yes	

**Table 3:** Pathogenic mutations were identified in the experimental lung cancer cohort.

Gene	No. Pathogenic Mutations/Gene	Mutation	Amino Acid Change	NSCLC		SCLC (*n* = 16)	Exon	Variance	Sample
				LUAD (*n* = 8)	LUSC (*n* = 8)				TT/Blood
ABL1	1	c.992A>G	p.Asn331Ser	-	-	1	6	Missense	1/1
CDKN2A	1	c.220G>T	p.Asp74Tyr	-	1	-	2	Missense	1/0
CTNNB1	1	c.136C>G	p.Leu46Val	1	-	-	3	Missense	1/0
ERBB2	1	c.2329G>A	p.Val777Met	-	-	1	20	Missense	1/0
IDH2	1	c.419G>A	p.Arg140Gln	1	-	-	4	Missense	1/0
KIT	2	c.1621A>C	p.Met541Leu	1	1	2	10	Missense	1/1
		c.1588G>A	p.Val530Ile	1	-	-	10	Missense	0/1
NRAS	2	c.182A>G	p.Gln61Arg	-	1	-	3	Missense	1/0
		c.182A>T	p.Arg140Gln	-	-	1	3	Missense	1/0
PIK3CA	3	c.1624G>A	p.Glu542Lys	-	-	2	10	Missense	1/0
		c.3196G>A	p.Ala1066Thr	1	1	4	21	Missense	1/0
		c.328G>A	p.Glu110Lys	-	-	1	2	Missense	1/0
PTEN	1	c.388C>T	p.Arg130Ter	-	-	1	5	Nonsense	1/0
RB1	1	c.409G>T	p.Glu137Ter	-	-	1	4	Nonsense	1/0
RET	1	c.409G>T	p.Ser649Leu	-	-	1	11	Missense	0/1
SMAD4	2	c.546C>G	p.Ile182Met	-	-	1	5	Missense	1/0
		c.1081C>T	p.Arg361Cys	-	-	1	9	Missense	1/0
STK11	1	c.465-1G>T	-	-	-	1	4	Unknown	1/0
TP53	23	c.1000G>T	p.Gly334Trp	1	-	-	10	Missense	1/0
		c.1001G>T	p.Gly334Val	1	-	-	10	Missense	1/0
		c.1036G>T	p.Glu346Ter	-	-	1	10	Nonsense	1/0
		c.215C>G	p.Pro72Arg	5	4	12	4	Missense	1/1
		c.400T>A	p.Phe134Ile	-	-	1	5	Missense	1/0
		c.461G>T	p.Gly154Val	-	-	1	5	Missense	1/0
		c.472C>G	p.Arg158Gly	-	-	1	5	Missense	1/0
		c.473G>T	p.Arg158Leu	-	1	-	5	Missense	1/0
		c.500A>G	p.Gln167Arg	1	-	-	5	Missense	0/1
		c.514G>T	p.Val172Phe	-	1	-	5	Missense	1/0
		c.559+1G>T	-	1	-	-	5	unknown	1/0
		c.575A>G	p.Gln192Arg	-	1	-	6	Missense	1/0
		c.713G>T	p.Cys238Phe	-	-	1	7	Missense	1/0
		c.722C>T	p.Ser241Phe	-	-	1	7	Missense	1/0
		c.725G>T	p.Cys242Phe	-	1	-	7	Missense	1/0
		c.742C>T	p.Arg248Trp	-	-	1	7	Missense	1/0
		c.797G>T	p.Gly266Val	-	-	1	8	Missense	1/0
		c.817C>A	p.Arg273Ser	1	-	-	8	Missense	1/0
		c.832C>T	p.Pro278Ser	-	-	1	8	Missense	1/0
		c.850A>G	p.Thr284Ala	1	-	-	8	Missense	1/0
		c.856G>A	p.Glu286Lys	-	1	-	8	Missense	1/0
		c.872A>G	p.Lys291Arg	-	-	1	8	Missense	1/0
		c.880G>T	p.Glu294Ter	-	1	1	8	Nonsense	1/0

**Table 4:** Overall patient survival status in the TCGA lung cancer datasets.

	Datasets TCGA	LUAD		LUSC		SCLC	
Gene		Altered/Unaltered	Logrank Test *p*-Value	Altered/Unaltered	Logrank Test *p*-Value	Altered/Unaltered	Logrank Test *p*-Value
ABL1		3/218	0.590	5/170	0.286	1/109	0.729
CDKN2A		55/166	0.0576	79/96	0.0531	1/109	0.359
CTNNB1		9/212	0.791	5/170	0.140	0/110	-
ERBB2		12/209	0.835	9/166	0.977	1/109	0.240
IDH2		4/217	**0.0243**	5/170	0.266	0/110	-
KIT		9/212	0.976	15/160	0.360	7/103	0.528
NRAS		7/214	**4.558 × 10^−4^**	6/169	0.122	0/110	-
PIK3CA		19/202	0.793	105/70	0.938	3/107	0.574
PTEN		6/215	0.641	30/145	0.283	7/103	0.441
RB1		17/204	0.910	18/157	0.418	80/30	0.410
SMAD4		11/210	0.480	10/165	0.882	2/108	0.112
STK11		42/179	0.412	6/169	**0.0403**	1/109	**0.0272**
TP53		103/118	0.173	142/33	**0.0419**	94/16	0.705

**Table 5:** Correlation coefficients (r) for lung cancer patients between the following criteria: alterations in TP53, KIT, PIK3CA, and STK11, tumor cancer stage, tumor size, lymph node involvement, metastatic spread and time to event (death).

	Altered TP53	Altered KIT	Altered PIK3CA	Altered STK11	Stage	Tumor Size	Lymph Node Metastasis	Distant Metastatic Spread	Adenocarcinoma	Squamous Cell Carcinoma	Small-Cell Lung Cancer	Death (Days)
altered TP53		0.138409	0.148581	0.185695	0.127563	0.267435	−0.06459	0.148581	−0.0619	−0.0619	0.107211	0.093676
altered KIT	0.1384091		0.03253	−0.04969	0.074796	−0.00678	0.046953	0.060351	0.019821	0.019821	−0.03452	0.049947
altered PIK3CA	0.1485808	0.03253		0.218218	0.055228	0.217574	0.22771	−0.01587	**−0.50918**	0.218218	0.251976	−0.03286
altered STK11	0.1856953	−0.04969	0.218218		0.108465	0.110783	0.248452	0.218218	0	−0.16667	0.144338	0.056454
Stage	0.1275627	0.074796	0.055228	0.108465		−0.10394	−0.04296	0.96862	0	−0.14548	0.125988	−0.00042
Tumor size	0.2674354	−0.00678	0.217574	0.110783	−0.10394		0.06877	−0.08411	−0.10585	0.052926	0.045835	−0.04025
Lymph node metastasis	−0.06459093	0.046953	0.22771	0.248452	−0.04296	0.06877		−0.00216	−0.19687	−0.03937	0.204598	0.113633
Distant metastatic spread	0.1485808	0.060351	−0.01587	0.218218	**0.96862**	−0.08411	−0.00216		−0.01827	−0.1644	0.158193	−0.00042
Adenocarcinoma	−0.06189845	0.019821	**−0.50918**	0	0	−0.10585	−0.19687	−0.01827		−0.33333	−0.57735	0.081326
Squamous cell carcinoma	−0.06189845	0.019821	0.218218	−0.16667	−0.14548	0.052926	−0.03937	−0.1644	−0.33333		−0.57735	−0.0643
Small-cell lung cancer	0.1072113	−0.03452	0.251976	0.144338	0.125988	0.045835	0.204598	0.158193	−0.57735	**−0.57735**		−0.01636
Death (days)	0.09367601	0.049947	−0.03286	0.056454	−0.00042	−0.04025	0.113633	−0.00042	0.081326	−0.0643	−0.01636	
deceased		−0.01491			0.085205	−0.10635	−0.05723	0.085205	−0.09343	0.036155	0.051473	−0.63572
Active smoker	−0.1072113	−0.20179	0	0.144338	−0.06299	−0.1375	0.068199	−0.03164	−0.28868	0.216506	0.0625	−0.24329
Former smoker	−0.1034483	−0.13566	**0.364698**	0.185695	−0.1756	0.029484	−0.18279	−0.16621	**−0.30949**	0.061898	0.214423	−0.14787
never smoker	0.1034483	0.135656	**−0.3647**	−0.1857	0.175596	−0.02948	0.182794	0.166209	**0.309492**	−0.0619	−0.21442	0.147874

Strength of association (r): 0.1–0.3 small; 0.3–0.5 medium; 0.5–1.0 large.

## Data Availability

Data are contained within the article or supplementary material.

## Supplementary Materials

The following supporting information can be downloaded at: https://www.mdpi.com/article/10.3390/jpm12030453/s1, Supplementary File S1 (full table with all the alterations identified in the NGS experiment on separate sheets for the alterations in tumor tissue samples (sheet 1—tumor tissue and sheet 2—blood).
